# Feasibility of integrating vestibular rehabilitation and cognitive behaviour therapy for people with persistent dizziness

**DOI:** 10.1186/s40814-019-0452-3

**Published:** 2019-05-20

**Authors:** Lene Kristiansen, L. H. Magnussen, B. Juul-Kristensen, S. Mæland, S. H. G. Nordahl, A. Hovland, T. Sjøbø, K. T. Wilhelmsen

**Affiliations:** 1 Department of Health and Functioning, Faculty of Health and Social Sciences, Western Norway University of Applied Sciences, P.O. box 7030, 5020 Bergen, Norway; 2 Department of Sports Science and Clinical Biomechanics, University of Southern Denmark, Odense, Denmark; 3 Norwegian National Advisory Unit on Vestibular Disorders, Department of Otorhinolaryngol and Head Neck Surgery, Haukeland University Hospital, Bergen, Norway; 4 Department of Clinical Medicine, University of Bergen, Bergen, Norway; 5 Solli District Psychiatric Centre (DPS), Nesttun, Norway; 6 Department of Clinical Psychology, University of Bergen, Bergen, Norway

**Keywords:** Dizziness, Persistent dizziness, Rehabilitation, Vestibular rehabilitation, Cognitive behaviour therapy, Gait velocity, Dizziness handicap inventory

## Abstract

**Purpose:**

To evaluate the feasibility of integrating vestibular rehabilitation and cognitive behaviour therapy (VR-CBT) for people with persistent dizziness in primary care.

**Design:**

Prospective single-group pre- and post-test study.

**Participants:**

Adults (aged 18–70) with acute onset of dizziness and symptoms lasting a minimum 3 months, recruited from Bergen municipality.

**Methods:**

Participants attended eight weekly group sessions of VR-CBT intervention. Feasibility outcomes consisted of recruitment and testing procedures, intervention adherence, and participant feedback, besides change in primary outcomes. The primary outcomes were Dizziness Handicap Inventory (DHI) and preferred gait velocity.

**Results:**

Seven participants were recruited for the study. All participants completed the pre-treatment tests, five participants completed the intervention and answered post-treatment questionnaires, and three completed post-treatment testing. Of the five participants, three attended at least 75% of the VR-CBT sessions, and two 50% of the sessions. Participants reported that the VR-CBT was relevant and led to improvement in function. DHI scores improved beyond minimal important change in two out of five participants, and preferred gait velocity increased beyond minimal important change in two out of three participants.

**Conclusion:**

The current tests and VR-CBT treatment protocols were feasible. Some changes are suggested to optimise the protocols, before conducting a randomised controlled trial.

**Trial registration:**

NCT02655575. Registered 14 January 2016—retrospectively registered

## Background

Dizziness is a common complaint in the general population, and the symptom has been linked to different aetiologies, with vestibular, psychiatric, and cardiovascular diagnoses being the most common [1]. Dizziness as a symptom in patients with vestibular disorders may persist [2, 3] and be accompanied by reduced balance, altered gait velocity, musculoskeletal tension and pain, and psychological complaints, such as anxiety and depression [4–9].

Vestibular rehabilitation (VR) is an exercise-based treatment recommended for people with unilateral vestibular disorders [10]. VR aims at reducing perceived dizziness and improve balance. The exercises are based on principles of habituation and adaptation/substitution, in addition to balance retraining [11], with exposure to a dizziness-provoking stimulus as core of the intervention. Home-based exercise programs are central in VR and promoted through information and dialogue [11]. In traditional VR, the attention is on challenges caused by the vestibular system [10]. However, the treatment also contains behavioural elements normally present in treatment for anxiety disorders [12], while musculoskeletal problems are only briefly addressed in the literature [13, 14]. In order to address vestibular, musculoskeletal, and psychological aspects of persistent dizziness, a modified VR (group-treatment) was developed [9]. In the intervention, the traditional types of exercises were combined with elements from the body awareness approach embedded in the Norwegian psychomotor tradition to target musculoskeletal problems [15]. Further, by giving time for self-reflection as part of the exercises and by arranging a dialogue following the exercise component of the VR, psychological elements were also addressed. The overall aims in this modified treatment were similar to those in the traditional intervention, with effects of reduced dizziness and pain, improved body flexibility, and trunk control which had influence on balance during walking [9, 16]. However, the modified program was evaluated in a longitudinal study with no controls, the sample size was small, and only short-term effects were examined.

A core element in cognitive behaviour therapy (CBT) is exposure to situations that are challenging, as is also partly included in VR, and a combination of both VR and CBT has been suggested as treatment for persistent dizziness [12]. In a systematic review, four randomised controlled trials (RCTs) reported improvement in dizziness following treatment with CBT, combined with VR or relaxation techniques [17]. However, the sample sizes in these studies were small, and the one study evaluating long-term effects did not find significant lasting effects. A recent RCT evaluating a brief intervention CBT for patients with chronic subjective dizziness found reduced perceived dizziness and experienced handicap and safety behaviours [7], with sustained effects at 6 months follow-up [18]. However, there were no changes regarding depression, anxiety, and stress.

As described above, there are few RCTs evaluating VR in combination with CBT. The results seem promising, but the small sample sizes and shortage of long-term follow-up indicate a need for further research. This was the basis for developing a study to evaluate the integration of the two treatment concepts. A feasibility study was conducted in accordance with the Medical Research Council (MRC) guidelines [19] and as part of the preparation for a RCT. The intervention was based on a treatment concept consisting of the modified VR presented above [9], and the CBT for people with dizziness and panic anxiety [7].

The aim of this study was therefore to evaluate the feasibility of a group intervention integrating VR and CBT (VR-CBT), for participants with persistent dizziness, as part of the preparation for a RCT. The primary focus for this feasibility study was to evaluate whether the testing procedures were feasible, if the participants could complete the questionnaire packet, whether the VR-CBT manual was accepted amongst participants and therapists, and whether the participants could adhere to the treatment. Secondly, the aim was to evaluate changes in the primary outcomes following the intervention.

## Materials and methods

### Design

The current feasibility study was conducted in accordance with the recommendations for the development of new interventions [20] and reported according to the recommendations for feasibility and pilot trials [21]. It was designed as a prospective study with a one-group pre- and post-test design, focusing on feasibility parameters and participant and physiotherapist feedback, in addition to improvement in primary outcomes.

### Participants and setting

Participants were recruited from four selected general practitioner (GP) clinics and from physiotherapists in the municipality of Bergen, during 1 month in the autumn of 2015. Participants were eligible for inclusion if they were aged between 18 and 70 years with acute onset of dizziness, where the symptoms had persisted for at least 3 months, and where the dizziness was provoked or exacerbated by head movements. Exclusion criteria were a known non-vestibular cause of dizziness (e.g. neurological, cardiovascular), diseases where vigorous head movements were contraindicated (e.g. osteoporosis of the neck), active benign paroxysmal positional vertigo (BPPV) (positive positional testing during screening), morbus Ménières, vestibular schwannoma, and serious disease (e.g. terminal cancer, severe psychiatric diagnosis).

### Procedure

Potential participants contacted the project coordinator by e-mail or telephone.

Following the initial telephone screening, eligible candidates were invited to an onsite screening at the Western Norway University of Applied Sciences (HVL). The first meeting had a total timeframe of 2 h and comprised screening for inclusion, pre-treatment testing, and a single individual brief intervention vestibular rehabilitation (BI-VR) treatment session. Time allocations for the different elements were scheduled as follows: approximately 15 min for screening, approximately 40 min for pre-treatment physical tests, approximately 30 min for pre-treatment questionnaires, and a single session of BI-VR lasting approximately 35 min. After the first meeting at HVL, a VR-CBT intervention was offered. This comprised treatment sessions once a week, for 8 weeks, at an outpatient clinic in Bergen. Following the intervention, the participants completed post-treatment questionnaires (approximately 25 min), prior to the post-treatment physical testing at HVL (approximately 45 min). In addition, participants were invited to take part in an interview exploring their experiences from the testing and treatment procedures.

The physiotherapists running the VR-CBT group treatment were recruited via an open e-mail invitation, which was sent to all physiotherapists employed by the Bergen municipality. The included physiotherapists participated in a competency course (Table 1) led by a physiotherapist, clinical psychologists, and a physiotherapist specialised in Norwegian Psychomotor Physiotherapy (NPMP). They were introduced to VR and CBT as separate concepts, the treatment manual integrating the two concepts, and they practised skills through individual- and group-based activities. Following the first and second VR-CBT sessions, the physiotherapists were offered further mentoring with one clinical psychologist and a physiotherapist. After completing the intervention, the physiotherapists were asked to answer a small questionnaire about their experience with the competency course and the intervention.

**Table 1:** Overview of the competency course for the VR-CBT treatment manual

Topic	Time allocation	Content
Introduction	9.5 h	- Introduction to VR, CBT, and treatment manual
		- Individual- and group-based skill training using gaze stability, habituation, and balance
Preparation prior to VR-CBT treatment	3 h	- VR-CBT manual content of each treatment session
		- Skill training: role-play using CBT according to VR-CBT manual
Organised PT reflection between treatment sessions	1 h (each session)	- Mentoring with clinical psychologist after each of the first two VR-CBT sessions

*H* hours, *VR-CBT* group intervention integrating vestibular rehabilitation and cognitive behaviour therapy, *VR* vestibular rehabilitation, *CBT* cognitive behaviour therapy, *PT* physiotherapist

The study has been approved by the Regional Committee for Medical and Health Research Ethics (2014-00921) and is registered at https://clinicaltrials.gov/ct2/show/NCT02655575 (NCT02655575). All participants provided written informed consent prior to inclusion in the study.

### Interventions

#### Brief intervention vestibular rehabilitation

Brief intervention vestibular rehabilitation (BI-VR) was as a single treatment session led by the project coordinator. It was based on elements from traditional VR developed for patients with dizziness [22, 23], but adapted to a single session in line with the brief intervention model developed for patients with low back pain [24]. The purpose of BI-VR was to provide the participants with an understanding of their dizziness and give them practical advice on how to improve their daily functioning. Information about how the perception of dizziness could be reduced was combined with a program of vestibular home exercises [11]. The participants were encouraged to stay active and provoke dizziness in line with established recommendations [10, 25]. The information was reinforced by an information leaflet [26].

#### Vestibular rehabilitation and cognitive behaviour therapy

The vestibular rehabilitation and cognitive behaviour therapy (VR-CBT) manual was developed through the collaboration between physiotherapists (one specialised in NPMP) and clinical psychologists. The treatment was offered as a group intervention with eight weekly sessions. Each session lasted approximately 2 h and was managed by two physiotherapists. Following each session, the participants were asked to complete a home exercise sheet. The tasks varied slightly from week to week, but all home exercise sheets asked for a registration of outside walking and duration of this. In addition, they were asked to register selected daily VR exercises from a pre-prescribed exercise sheet from session number three onwards. The aim of the VR-CBT intervention was to address both physical and psychological challenges of dizziness. This was done by providing opportunities for the participants to practise exercises in a safe environment, accompanied by reflections on dizziness and safety and avoidance behaviours. In brief, the VR component included balance and body awareness training, habituation and adaptation exercises, and relaxation [6]. The exercises could be adapted to the individual, guided by a physiotherapist, and included, for instance, changes to the base of support, speed of movement, and environmental conditions. The CBT was based on a model used for anxiety and panic disorders [27–29], addressing catastrophic misinterpretations, safety and avoidance behaviours, and the fight or flight response, topics also covered in a previous study on people with persistent dizziness [7]. All sessions included both VR and CBT, with the first three sessions mostly emphasising CBT, while the subsequent five sessions mostly emphasised VR. This allowed the participants to use the CBT topics to reflect on balance strategies, movement patterns, and bodily reactions during the physical exercises.

### Outcomes

The outcomes included process-related outcomes and participant and physiotherapist feedback, in addition to changes in primary outcomes, as described in detail below.

#### Process-related outcomes

The outcomes included recruitment and adherence to the test and treatment protocols. The participants completed subjective outcomes (questionnaires) on a tablet, or online, allowing automated calculation of time spent on each questionnaire. The participants further completed objective outcomes (see below).

#### Participant and physiotherapist feedback

The main topics in the interviews were the participants’ experiences with the test protocol, the intervention, and perceived benefits of the intervention. The project coordinator, supported by either the project leader or a research assistant, facilitated the participant interviews. The questionnaire completed by the physiotherapists covered topics regarding experience and satisfaction with the competency course, the treatment protocols, and the VR-CBT intervention.

#### Subjective outcomes

The primary subjective outcome was change in the impact of dizziness on daily function and quality of life and was assessed using the Norwegian version of the Dizziness Handicap Inventory (DHI) [30, 31]. The DHI consists of 25 items, with a score range of 0–100 points [30], and higher scores indicating greater dizziness-related handicap. A cut-off for dizziness-related handicap is established at a minimum 29 points [31], and moderate handicap at 31–60 points [32]. Test-retest reliability of the Norwegian version is reported to be acceptable, and minimal important change (MIC) has been established to be 11 points [31].

Secondary subjective outcomes included the following: the Vertigo Symptom scale—short form (VSS) describes perceived severity of dizziness [33, 34], with acceptable test-retest reliability [35], and severe dizziness established at 12 points or more [33]. The Hospital Anxiety and Depression Scale (HADS) [36], the Body Sensation Questionnaire (BSQ) [37], and the Mobility Inventory of Agoraphobia-Alone (MI-A) [37] describe levels of anxiety, depression, panic-related symptoms, and avoidance behaviour. They have all shown satisfactory internal consistency and clinical cut-offs established (HADS 12 points, MI-A 1.65 points, and BSQ 2.31 points) [37–40]. A modified version of the Panic Attack Scale (PAS) [28] presents frequency and experienced severity of markedly increased bouts of dizziness. The EQ5D-5L [41] describes quality of life, with acceptable test-retest reliability established [42]. The Subjective Health Complaints (SHC) inventory reports incidents and extent of health complaints, with satisfactory internal consistency [43].

General demographic information included age, civil status, educational level, employment, and disease history.

#### Objective outcomes

The primary objective outcome was preferred gait velocity measured in meters per second (m/s) [44]. Participants walked, at their preferred velocity, along a 6-m pathway, with an additional 1-m start-up and slow-down stretch at each end. They were timed using a stopwatch, and the mean preferred gait velocity of two attempts was calculated. A change of 0.1 m/s is recognised as meaningful in older adults [45], and acceptable test-retest reliability has previously been established in people with dizziness [44].

Secondary objective outcomes included the following: the Timed Up-and-Go (TUG) [46] test evaluates the risk of falls with elements that may provoke dizziness, with acceptable test-retest reliability demonstrated [47]. The Dual Task Walking test (DTW) examines the effects of an arithmetic cognitive task on gait velocity comparing walking with and without an arithmetic task [48]. Clinical Dynamic Visual Acuity (CDVA) test evaluates gaze stability during head movements [49], with satisfactory reliability [50]. Perceived dizziness before and after head movements (HmDizz) [44] was evaluated using the numeric rating scale. Grip-strength evaluates muscle status [51], with acceptable reliability [52]. The Modified Clinical Test for Sensory Interaction and Balance (mCTSIB) test [53] and sharpened Romberg test [54] assess standing balance by timing the participants for 30 s, both with established acceptable test-retest reliability [54, 55]. Four items from the movement domain of the Global Physiotherapy Examination (GPE) evaluates musculoskeletal aberrations. The selected items were based on previous studies focusing on people with vestibular disorders [6, 9].

### Randomisation and blinding

The procedures for randomisation and blinding were not evaluated as no control group was used in the present feasibility study.

### Analyses

Demographic and process-related outcomes were registered, while the recorded participant feedback included in the interviews were transcribed and thoroughly read to get an overall impression of the material. Afterwards, all meaningful text units were identified and organised into categories related to assessment, intervention, and perceived benefit of the interventions.

Further, the physiotherapy questionnaire was analysed descriptively, and changes in the primary outcomes were reported in relation to minimal important change (MIC) [6, 56]. The secondary outcomes were evaluated as part of the feasibility of the total test protocol, and only pre-treatment results are presented.

## Results

Seven participants (mimicking the numbers planned in one VR-CBT treatment group) were included and completed all pre-treatment tests in the study. The participants (two men) had a mean age of 38 years (SD 8), ranging from 27 to 48 years. The dizziness complaints had been present from 6 to 503 months, with six out of seven participants presenting dizziness for 17 months or more. Five of these participated in the VR-CBT intervention and completed the questionnaires post-intervention. Three of these also completed post-treatment physical testing (Table 2) and took part in the additional interviews (two participants in a group interview and one in a personal interview) giving feedback related to their experiences during participation in the study.

**Table 2:** Pre- and post-treatment scores on primary outcomes, for participants with persistent dizziness (*n* = 7)

	Test	Participant							Mean
		1	2	3	4	5	6	7	
DHI (score 0–100, 100 = worst)	Pre	58	50	76	54	58	60	54	56
	Post	46	52			36	60	46	48
	Diff	− 12	2			− 22	0	− 8	8
Preferred gait velocity (m/s)	Pre	1.01	0.99	1.06	1.12	0.82	1.18	1.36	1.08
	Post		1.73			0.97		1.31	1.34
	Diff		0.74			0.15		− 0.05	0.28

*DHI* Dizziness Handicap Inventory, *Pre* pre-treatment test, *Post* post-treatment test, *Diff* difference

### Process-related outcomes

#### Participant recruitment and study adherence

**Fig. 1:**
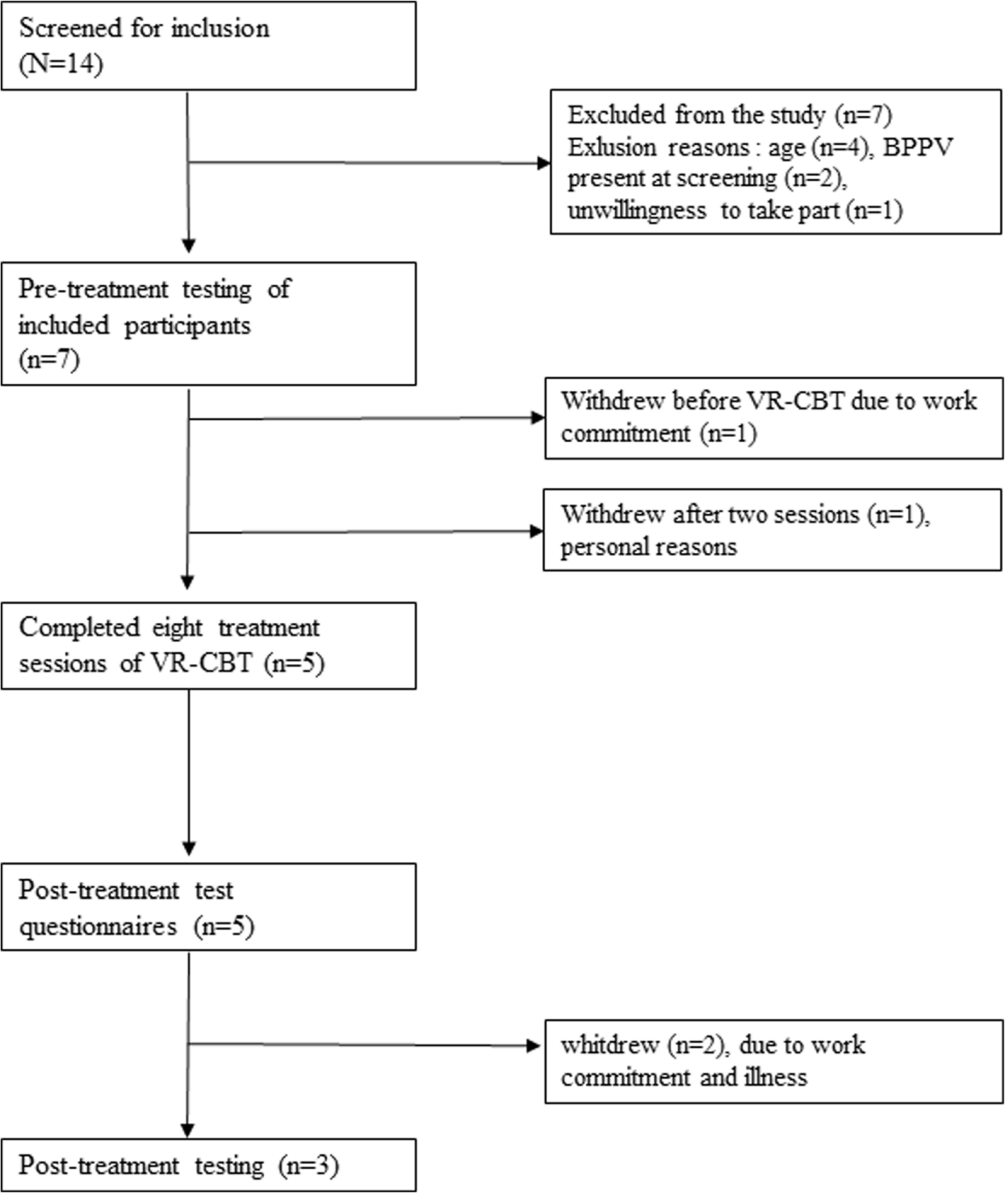
Flow chart for recruitment, inclusion, treatment, and follow-up of participants in feasibility study

In 1 month, 14 people with persistent dizziness were screened, and seven participants were included in the study (Fig. 1). Reasons for exclusion were age outside acceptable age range, positive BPPV at screening, and unwillingness to participate. Two participants withdrew from the study before or during the initial sessions of the VR-CBT intervention due to time constraints and personal reasons. Of the five participants completing VR-CBT and post-treatment outcomes, two participants failed to complete post-treatment physical testing, due to unspecified illness and work commitments.

#### Test duration

Screening for inclusion lasted 15–25 min, and physical testing lasted 25–40 min. The participants spent a mean time of 36 min (range 25–53 min) to complete the pre-treatment questionnaires, and three participants spent a mean time of 25 min (9–50 min) to complete the post-treatment questionnaires.

#### Test protocol and outcomes

All seven participants were able to complete the total pre-treatment test protocol. However, missing values were seen in MI-A, the PAS, and the EQ5D-5L VAS (Table 3).

**Table 3:** Pre-treatment scores on secondary outcomes, for participants with persistent dizziness (*n* = 7)

	Participant							Mean
	1	2	3	4	5	6	7	
Subjective outcomes								
VSS (0–60, 60 = worst)	35	19	10	23	30	24	23	23
HADS (0–42, 42 = worst)	15	10	11	13	5	13	13	11
SHC total (0–78, 78 = worst)	26	16	21	25	27	26	17	23
Modified PAS								
Frequency (0–4, 4 = worst)	4	0	0	2	0	4	0	1
Severity (0–8, 8 = worst)	4	0	6	6		6		4
BSQ (1–5, 5 = worst)	1.47	2.24	2.41	1.53	1.88	2.00	1.41	1.85
EQ5D-5L index (− 0.21–1, 1 = best)	0.35	0.68	0.54	0.68	0.61	0.64	0.64	0.59
EQ5D-5L (0–100, 100 = best)	40	49	35	50	29	40	50	42
MI-A (1–5, 5 = worst)	2.44	1.89	1.81	2.26	1.41	2.85		2.11
Objective outcomes								
TUG (sec)	5.3	5.0	6.3	4.2	10.5	4.7	4.6	5.8
Dual task walking (m/s)								
Preferred	1.0	1.1	1.0	1.1	0.7	1.2	1.2	1.0
Cognitive	0.8	0.8	1.0	1.0	0.3	0.6	1.0	0.8
CDVA (change score)	1	−1	0	0	1	0	1	0
HmDizz (NRS 0–10, 10 = worst)								
Head stationary	5	6	3	3	1	7	3	4
After head oscillations	7	10	6	7	4	10	7	7
Grip strength right (kg)	43.2	37.7	20.3	30.1	22.4	22.0	29.8	29.4
Grip strength left (kg)	40.2	27.6	14.9	27.4	24.0	18.6	32.7	26.5
mCTSIB (0–30 s, 30 = best)	30.0	30.0	30.0	30.0	30.0	30.0	24.1	29.2
Sharpened Romberg (0–30 s, 30 = best)								
Eyes open	30.0	30.0	30.0	30.0	30.0	30.0	30.0	30.0
Eyes closed	14.0	9.0	30.0	22.3	29.3	25.2	3.0	19.0
Elements of GPE (score − 2.3 to + 2.3, 0 = best)								
Lumbo-sacral flexion	2.0	1.0	1.3	1.0	2.0	1.3	1.3	1.4
Head-neck flexion	2.0	1.3	2.0	0.0	1.3	2.0	1.7	1.5
Shoulder retraction	− 1.0	2.0	1.3	− 0.3	1.3	− 0.7	1.0	1.1
Elbow drop	1.7	2.0	0.3	0.3	0.3	0.0	0.0	0.7

*VSS* Vertigo Symptom Scale, *BSQ* Body Sensation Questionnaire, *MI-A* Mobility Index—alone, *PAS* Panic Attack Scale, *HADS* Hospital Anxiety and Depression Scale, *SHC* Subjective Health Complaints, *TUG* Timed Up-and-Go, *CDVA* Clinical Visual Dynamic Acuity test, *HmDizz* head movement induced dizziness, *NRS* numeric rating scale, *mCTSIB* modified test of interaction and balance, *sec* seconds, *GPE* Global Physiotherapy Examination

#### Adherence to the VR-CBT treatment

Of the five participants attending the VR-CBT treatment, two attended seven sessions (88%), one participant six sessions (75%), and two participants four sessions (50%). Reasons for not attending were dizziness-related illness (*n* = 3), unspecified illness (*n* = 4), and other personal reasons (*n* = 5). As two participants missed session number two and one was unable to attend session number three, short booster sessions were offered prior to the next sessions, to ensure that CBT topics were covered amongst all participants.

Nineteen out of 25 home exercise sheets were completed (six were missing due to VR-CBT absence), and the participants registered daily VR exercises in 80% of these cases. In addition, four out of five participants walked outside at least three times a week for a duration of 30 min or more.

### Participant and physiotherapist feedback

#### Physiotherapy competency course

Both physiotherapists reported that the course was well organised with relevant lectures and teaching scenarios. The reflection sessions with the psychologist following the first treatment sessions were likewise perceived as helpful in order to meet the participants in the best manner.

#### Test protocol

The participants reported that the test protocol was relevant to their complaints. However, they perceived the objective tests as physically demanding and tiring, and completing the questionnaires as time-consuming.

#### VR-CBT intervention

The participants appreciated the VR-CBT intervention. One participant expressed it was useful to have mostly CBT in the first three sessions, and more emphasis on the exercises in later sessions, while another expressed that the treatment was more appropriate when the focus shifted to exercises and progression of these. One participant felt that CBT focused too much on psychological challenges. All three participants reported that the exercises initially were very hard and that they felt dizzy and nauseous after the sessions. They also expressed that during the program, they each learned to adjust the exercise intensity levels and that they experienced progress. The relaxation exercises at the end of each session were further perceived as beneficial. One participant commented that it was difficult to set aside time to do the home exercises, while another found it easy to incorporate them into the daily routines.

In the physiotherapy feedback, one reported that the intervention and exercise progression seemed appropriate for the population and that more exercises could have been incorporated. The other physiotherapist felt that the participant group was heterogeneous, with some participants progressing slower than others, making exercise adjustments necessary. Both reported that the treatment manual was easy to follow and individually adjust where necessary.

Each session was set to last 2 h. However, the physiotherapists felt that some of the participants had reduced exercise ability, and the full 2 h were not always utilised.

#### Group dynamics

The participants felt relaxed being in a group setting, they liked meeting other participants with similar problems, and they reported an increased motivation when performing exercises together compared to exercising alone (home exercises). One person wished there was more time to share experiences related to dizziness.

#### Self-reported benefit of intervention

Overall, the participants reported improvement in function after VR-CBT. The CBT made them more aware of how they moved and which strategies they used that were not beneficial. They reported that after the treatment, they were more active and had incorporated VR exercises into their daily routines. One participant reported, “I feel that the dizziness is not as prominent in my life as before. I can be active for longer, it is easier to focus at work, and I do not need as many breaks anymore. I have a positive outlook for the future, and if I keep doing the exercises I believe I can get even better.”

### Outcomes

#### Primary outcomes

At post-treatment outcomes, two participants reported decreased DHI scores beyond MIC (12 and 22 points), and two participants increased their preferred gait velocity beyond MIC (0.74 m/s and 0.15 m/s) (Table 2).

#### Secondary outcomes

Most of the participants responded as expected at pre-treatment testing, and the selected subjective outcomes were found relevant for the condition of persistent dizziness. This included severe dizziness (VSS > 12 points), experienced avoidance behaviour when alone (MI-A > 1.65 points), and daily dizziness related attacks on the PAS*.* Participants also presented with slower walking when adding a cognitive task, non-completeness of the sharpened Romberg with eyes closed, and GPE scores indicating deviations from the norm on most tests. In addition, they reported increased dizziness complaints after 1 min of head oscillations (Table 3).

## Discussion

The recruitment and adherence to the study were satisfactory. All participants could complete the objective and subjective outcomes, both participants and physiotherapists reported testing and treatment as relevant, and participants improved on primary outcomes. However, some changes to test protocol and secondary outcomes were necessary, as described below.

Recruitment to the study seemed feasible. Seven participants were included during the month-long recruitment period, through outpatient physiotherapists and four out of the existing 73 GP clinics. With 864 people on sick leave due to dizziness or reduced balance in the county^1^, the recruitment potential was anticipated to be satisfactory for a larger scale study. It was believed that information to all GP clinics and the public (through newspaper advertisements and social media) would ensure adequate recruitment for the planned RCT. The completion rate (71%) in the present study was slightly lower than other studies on similar populations, which varied from 80 to 100% [7, 16, 33, 57]. In addition, two of the participants did not complete all elements of the post-treatment testing. Missing post-treatment tests have also been reported previously [33]. The recruitment and completion rate in this study, supported by previous research, were important considerations when establishing the sample size and recruitment time for the planned RCT.

It was difficult to complete the test protocols and BI-VR within the set time frame. As a result, the following adjustments of the protocols and administration of the BI-VR were performed. Since gait assessments comprised three tests (preferred gait velocity, TUG, and DTW) and two of these (preferred gait velocity and TUG) are reported as predictive of falls [58], one (TUG) could be omitted. This was because none of the participants reached the cut-off levels for falls at TUG. (11.1 s) [46]. Further, as the DTW was not able to measure counting strategies, the test protocol was adjusted to accommodate this. To further reduce the testing duration, one of the two tests for evaluating standing balance (sharpened Romberg) was omitted. As the other test (mCTSIB) demonstrated ceiling effect and a limited ability to measure balance strategy (sway), the test protocol was altered to include quantification of sway with a force platform.

Further, due to time limitation in the feasibility test protocol, the BI-VR treatment was removed from the first meeting, and administered as a separate 1-h appointment, run by a physiotherapist not involved in the data collection.

All participants were able to complete the questionnaires but most spent more time than expected. The missing values on the PAS could suggest that the participants did not understand the questions or that the items were perceived as irrelevant. In order to ensure data completion, the participants were allowed to complete the questionnaire on paper, with support from the tester.

The participants reported that the outcomes included seemed appropriate, and many of the outcomes utilised in this study have previously been used in populations with dizziness [2, 5, 7, 9, 32, 38, 46, 59, 60]. However, during the interviews, the participants reported that they tired quickly during treatment and that the testing was demanding. In addition, the physiotherapists commented that participants fatigued in the VR-CBT sessions. This was a dimension not covered by the present outcome measures, and to the authors’ knowledge, only one study with vestibular participants has reported this previously [61]. Despite the possible increased strain of an added questionnaire, it was deemed as important to incorporate this element amongst the outcomes, and fatigue was added as a subjective outcome.

Since all the participants were able to complete the questionnaires, the Agoraphobic Cognition Questionnaire (ACQ), part of the standard packet used in panic anxiety research together with the BSQ [29, 40], was added to the questionnaire packet to allow for a more comprehensive assessment of panic-related symptomatology in the study population.

The VR-CBT seemed appropriate for both participants and physiotherapists. Although the participants had similar characteristics, they could be described as a heterogeneous group, as commented on by one of the physiotherapists. This seemed to be accommodated for in the intervention manual, which allowed individual exercise adaptation and progression, which has also been suggested previously [62]. The participants were encouraged to pace themselves, but at the same time, the current program progressed with participants being able to take more control over the exercise intensity themselves, as commented by some of the participants.

Even though one person commented that it was difficult to set aside time to do home exercises, these exercises were considered a central part of VR and were therefore kept in the treatment program.

The participants improved on primary outcomes (reduced DHI scores and increased gait velocity) following the VR-CBT, and they reported being more active with the dizziness being less prominent in their lives. The improved primary outcomes have also previously been found in effect studies of only VR, only CBT, or a combination of the two [7, 63].

Most of the participants’ pre-treatment scores were equivalent to those in other studies [5, 7, 9, 32, 38], however, with some exceptions [2, 46]. In addition, the current study also included outcomes used in other population, but not used in this population before [28, 43, 48, 51].

In summary, small changes were made to the test-protocol, a few outcomes were removed, and others added. In addition, one adjustment was made to the VR-CBT protocol, by reducing the initial exercise intensity, allowing participants more time to adjust to the exercises.

The study has some limitations. The small sample size and the absence of a control group mean that the changes in primary outcomes cannot be used to evaluate the effectiveness of the intervention. Another possible weakness is the risk of bias due to lack of feedback from those who did not complete all aspects of the study. However, the interviewed participants gave both positive and negative feedback, which was also supported by the physiotherapists. Therefore, as in similar feasibility studies, the current process-related outcomes and participant and physiotherapist feedback constitute the main results, on which to build future RCTs. The strengths are the standardised test procedures and interventions and the use of reliable and valid outcome measures used as main outcomes in the study.

## Conclusion

The current test and VR-CBT treatment protocols were feasible. However, some changes are suggested to optimise the protocols, before conducting a randomised controlled trial.

## Abbreviations

ACQ: Agoraphobic Cognition Questionnaire
BI-VR: Brief intervention vestibular rehabilitation
BPPV: Benign paroxysmal positional vertigo
BSQ: Body Sensation Questionnaire
CBT: Cognitive behaviour therapy
CDVA: Clinical Dynamic Visual Acuity
DHI: Dizziness Handicap Inventory
DTW: Dual Task Walking test
GP: General practitioner
GPE: Global Physiotherapy Examination
HADS: Hospital Anxiety and Depression Questionnaire
HmDizz: Perceived dizziness before and after head movements
HVL: Western Norway University of Applied Sciences
mCTSIB: Modified Clinical Test for Sensory Interaction and Balance
MI-A: Mobility Inventory of Agoraphobia—Alone
MIC: Minimal important change
MRC: Medical Research Council
NPMP: Norwegian Psychomotor Physiotherapy
PAS: Panic Attack Scale
RCT: Randomised controlled trial
SD: Standard deviation
SHC: Subjective Health Complaints
TUG: Timed Up and Go
VAS: Visual analog scale
VR: Vestibular rehabilitation
VR-CBT: Integration of vestibular rehabilitation and cognitive behaviour therapy
VSS: Vertigo Symptom Scale—Short form

## Footnotes

1 Statistics obtained from NAV, nav.statistikk@nav.no on 13.10.15
